# The Shifting ‘Self’ of Science’s Self-Governing Capacity: Four Decades of Research Integrity Discussions in *Science* and *Nature*

**DOI:** 10.1177/03063127251392603

**Published:** 2025-11-07

**Authors:** Ulrike Felt, Florentine Frantz

**Affiliations:** 1 University of Vienna, Austria

**Keywords:** research integrity, misconduct, self-correction of science, narrative choreographies, frictions

## Abstract

The belief in science’s inherent self-corrective nature has been challenged by growing concerns over research integrity violations, leading to heightened scrutiny of scientific processes. Examining 40 years of discussions in *Science* and *Nature*, this article explores how debates on issues around research integrity reflect important shifts in the very meaning of ‘scientific self’ when speaking about the self-corrective capacity of science as well as evolving ‘geographies of responsibility’ within the research system. These journals—key voices in science and policy discourse—offer a lens to explore these processes of gradual transformation. We identify key narrative threads and turning points in understanding who is accountable for ensuring research integrity—extending beyond individual researchers to include institutions, funding bodies, journals, and whistleblowers. The analysis highlights how the scientific community has progressively reassembled its self-image, adapting to complex systemic challenges while engaging diverse stakeholders. These narratives, we argue, do more than document instances of transgressions of good scientific practice: They map broader transformations in the research ecosystem, revealing changing values, roles, and expectations. By analysing these shifts, we offer new insights into the interconnections between integrity concerns and systemic change, and into the conditions necessary for fostering responsible research practices and sustaining (public) trust in science.

[R]eassurances that there is nothing to worry about—because the scientific process is self-corrective, and that mistakes, deliberate or accidental, will be found out sooner rather than later—are no longer enough. [Nature, 1986^1^]

Retractions are part of science, but misconduct isn’t. … Journals, funders and institutions that employ researchers all want to produce or disseminate rigorous scientific knowledge—and all can learn lessons from misconduct cases. [Nature, 2024^2^]

The comforting notion that the scientific process is inherently self-corrective and that errors will inevitably come to light has been and still is a pervasive narrative. However, over recent decades, growing debates around violations of research integrity have started to challenge ‘assumptions about the efficacy of self-policing in the scientific community, often resulting in the imposition of new regulations, procedures and oversight bodies’ (Martin, 2013, p. 1105). As a result of the new visibility of these issues, journals now have their own publication ethics statements or are part of the Committee on Publication Ethics (COPE, n.d.). Highly visible international Codes of Conduct have been crafted (ALLEA, n.d.). Beginning with a meeting in Lisbon in 2007, the World Conference on Research Integrity (WCRIF, n.d.) has been co-organized by the European Science Foundation and the US Office of Research Integrity. Together, all this can be taken as an indicator of the growing awareness that both principles and practices of research integrity need more attention. Yet, it remains unclear how to tackle the issue in practice (Felt, 2015).

The push for more open debates has been driven by a range of concerns, including the growing perception of an increase in academic misconduct, the rising number of retracted papers (even though retraction itself is not viewed as inherently problematic), evident flaws in the peer review process, the undue influence exerted by those funding or conducting studies on their outcomes, and, more recently, the issues encapsulated by the term ‘reproducibility crisis’. Also, in the field of social science research, we now find a considerable body of studies around transgressions of good scientific practice that have delivered valuable insights into the dynamics at work in contemporary research. Besides a growing activity of researchers expressing personal experiences and perspectives in journal commentaries and blogs, we observe more structured analytical interests engaging in studying researchers’ perspectives (Olesen et al., 2018). We encounter studies investigating how contemporary academic incentive structures might tacitly foster unethical actions (Edwards & Roy, 2017), what is made (in)visible when misconduct cases are identified (Hesselmann & Reinhart, 2021), looking into research culture and practice (Valkenburg et al., 2021), questions of publication ethics (Jacob, 2019), the influence of public and private organizations on norms of research integrity (Montgomery & Oliver, 2009) and many more. These accounts highlight the diversity of practices that researchers consider and point to the influence research environments have on the capacity to produce knowledge effectively (Felt, 2025a; Haven et al., 2020).

In this article, we do not aim to study specific forms of transgressions of research integrity or concrete cases of transgressions; instead, we use debates on research integrity as a lens to explore evolving ‘geographies of responsibility’—a concept adapted from Akrich (1992)—within the research system. To do so, we use the key scientific journals, *Science* and *Nature*, as two platforms where these debates can be followed *in* their respective time and *over* several decades. *Nature* and *Science* are highly ranked multidisciplinary journals and have been a core stage for significant scientific insights since the 19th century (Ding et al., 2018). Both of them also have a long tradition reflecting on broader scientific developments as well as on science-policy and science-society relationships and shaping the idea of scientific publishing (Baldwin, 2015). Studying articles from the past 40 years in these two outlets allows us to understand how these issues were reflected upon in their respective time and it teaches us about the value-related transformations that occurred over this period.

Analysing articles that discuss fraud, misconduct, reproducibility issues, and research integrity over an extended period allows us to trace shifting understandings of who bears responsibility for identifying, assessing, defining, regulating, correcting, or managing such issues. Central to our approach is what we call the ‘scientific self’—the reflexive figure invoked in repeated claims about science’s capacity for ‘self-correction’ or ‘self-regulation’. This is not simply an abstract ideal, but a historically contingent self-image of science, continuously reassembled in response to emerging challenges and crises. The ‘scientific self’ integrates memories of past achievements and failures, articulates present responsibilities, and projects desired futures for the scientific enterprise.

For instance, the 1975 Asilomar Conference on recombinant DNA technology—organized by scientists themselves to formulate guidelines on acceptable research practices—stands as a vivid example of a moment when ‘the authority of a scientific community to constitute itself, to predict possible futures, and to define responsibilities for them in the present’ (Hurlbut, 2015, p. 126) became visible. It was an instance in which the ‘scientific self’ took explicit collective form, exercising its perceived authority to govern its own conduct.

Our analysis, therefore, seeks to identify when and how such scientific selves are constituted, what roles they assume in taking responsibility for research integrity, and how their constitution shifts over time, gradually enlarging this self that has to care for integrity issues. Staying with the notion of ‘scientific self’, even in the enlarged form, allows us to keep sight of a key claim that has long been central to science’s cultural authority: the idea that science is uniquely capable of governing itself. Over time and with wider transformations of society, this can only be achieved by allowing an extension of the self. When responsibilities are redistributed—say, when institutions or funders take on a larger role—the scientific self does not disappear; rather, it is extended, fragmented, and rearticulated.

We put narratives on centre stage as they allow us to externalize and share our experiences (Czarniawska, 2004); they ‘organize our experience and our memory’ (Bruner, 1991, p. 4) and find expression in the form of ‘stories, excuses, myths, reasons for doing and not doing, and so on’ (Bruner, 1991, p. 4). The two journals create narratives about transgressions of good practice, why they find them problematic, who is involved and who should act, how they conceptualize research and the institutional environment and, ultimately, how responsibility is or should be distributed and assumed. Identifying dominant issue-focused narrative strands addressing scientific misconduct and analysing their entanglement over time allows us to identify different models of the scientific self emerging in specific periods. As with every classification, the categories and the temporal periods we identify are ideal types and have, in practice, fuzzy boundaries. However, this periodization will show how, over time, the scientific self gradually assembles and integrates more stakeholders besides researchers, such as funding agencies, scientific journals, universities, or offices for research integrity, but also whistleblowers. This indicates a clear shift in changing understandings of what it means to be responsible for and to behave responsibly in research. Identifying these shifts can help us better understand how concerns about research integrity are deeply intertwined with transformations in the overall research system, allowing for a deeper grasp of the changing choreographies of values and actors.

Our findings offer a detailed analysis of how scientific misconduct discussions have revealed a shift from insular, self-regulating scientific ideals to a broader, multi-stakeholder approach involving institutional actors and formalized oversight. While these changes have improved accountability and public trust, they also highlight the need to move beyond compliance-focused models towards fostering a collaborative, care-oriented culture of integrity that aligns ethical practice with the evolving complexities of science and society.

## Capturing Transformations in Research Systems

During the period examined here, research systems have undergone significant changes, often linked to broader socio-political and economic shifts. These have involved new public management principles, and reshaping how research is organized, funded, and evaluated. These changes have had profound implications for scientific inquiry, researchers, and research institutions. Simultaneously, there has been a growing call for a new social contract between science and society (e.g., Nowotny et al., 2001), rethinking knowledge production through concepts such as responsible research and innovation (Stilgoe & Guston, 2017).

Gradually, and varying in intensity and speed in different national contexts, scientific research has become increasingly subject to market-like principles, prioritizing efficiency, competition, and measurable outcomes. This has led to a shift in the ‘orders of worth’ (Stark, 2009) guiding research, along with new governance structures and performance-oriented priorities (Fochler et al., 2016), installing various forms of epistemic capitalism (Fochler, 2016). Public funding is often tied to demonstrating societal impact, emphasizing relevance, and commercial potential (Hessels et al., 2009).

Pressures for accountability and excellence have driven the introduction of performance metrics that shape competition in the research system (Shore & Wright, 2015; Strathern, 2000) and created temporal pressures in many parts of academia (Felt, 2025a). These metrics—focused on outputs like publications, citations, patents, and commercialization—valorize quantifiable activities, sidelining other forms of scholarly work. The emphasis on efficiency and productivity has significant governance effects, influencing institutional practices and individual behaviours (Espeland & Sauder, 2007). As a result, research is increasingly viewed as an economic and competitive endeavour, rather than a purely intellectual or societal pursuit.

In parallel, there has been a rise in ‘projectification’, with research funding typically allocated through competitive grants for specific projects with clear outcomes, timelines, and deliverables (Ylijoki, 2015). This trend makes long-term, exploratory research harder to fund, as researchers must continually apply for short-term grants, focusing on practical results within limited timeframes. This shift undermines career stability as reliance on short-term contracts grows. It also alters academic rhythms, with increased pressure to clarify the relationship between outputs and time invested (Felt, 2025a; Garforth & Cervinková, 2009). These conditions raise concerns about the effects on early-career researchers and the potential for compromized academic standards (Krstic, 2015).

Amidst these changes, the intensification of accountability—captured by the notion of the audit society (Strathern, 2000)—has brought an increased focus on control and planning. Gaining and maintaining public trust has become a critical issue. Researchers are expected to embrace the role of the academic entrepreneur, navigating the complex funding landscape while adapting to the logic of academic capitalism (Fochler, 2016; Shapin, 2008).

A closely entangled line of transformation concerns the evolving social contract between science and society, which defines the mutual responsibilities, roles, and expectations regarding the production and application of scientific knowledge (e.g., Nowotny et al., 2001). Science is increasingly seen as inseparable from societal needs, with growing demands for researchers to consider their work’s social, ethical, and political implications. This shift is embodied in concepts like Responsible Research and Innovation (Owen et al., 2012), which call for integrating ethical reflection, social engagement, and environmental sustainability into the research process. Researchers are now expected to ensure their work aligns with societal needs, anticipates potential harms, and involves stakeholders in shaping research agendas.

The new social contract also signals a shift in power dynamics within the research system. Traditionally, researchers and institutions held the dominant role in determining research priorities, with little input from the public. Today, there is a growing recognition that society—mediated through governments, funding bodies, and civil society—should have more influence in shaping scientific agendas. This shift is reflected in the increasing demands for accountability and a clearer demonstration of the societal relevance of research.

## Frictions as Our Locus of Analysis

Transgressions of good scientific practice are disruptive events that expose fault lines in an otherwise smooth-running system. Such moments provide valuable opportunities for STS researchers to study research cultures and practices (Valkenburg et al., 2021). Much like controversies, these moments make explicit otherwise-hidden assumptions about science’s norms and values, revealing the processes and socio-material networks that typically remain black-boxed. These disruptions allow us to scrutinize the complex forces shaping scientific facts and artefacts. Thus, controversial debates in journals should not be seen merely as informal assessments of science but as integral to the governance of research (Cambrosio & Limoges, 1991).

Such moments also serve as ideal spaces to observe the co-production of social and knowledge orders within research (Jasanoff, 2004). They offer a glimpse into how ideals of good scientific practice are shaped by the research systems in which they are embedded, while simultaneously influencing the role of science in contemporary society. Transgressions, therefore, are not just disruptions of an ideal state of science but a lens through which we can better understand the development and positioning of science in contemporary societies. These breaches of good practice are particularly concerning because they not only threaten public trust in science (Ampollini & Bucchi, 2020) but also disrupt the trust relations essential to the creation of scientific knowledge (Shapin, 1995, 2008).

In the *Science* and *Nature* articles we find that instances of misconduct are seldom portrayed as sites of openly visible conflicts; more often, they appear as moments of friction—subtle tensions where differing values, expectations, and practices meet. To capture this nuance, we make use of Tsing’s (2005) concept of ‘friction’, which offers valuable analytical traction. Tsing invites us to view friction not merely as an obstacle, but as a generative force—an opportunity to illuminate the dynamics, negotiations, and transformations that unfold when divergent worlds come into contact. In the context of research, frictions arise when individuals from diverse backgrounds navigate complex and often unequal interactions in shared environments. These moments of friction, where epistemic, institutional, and social contexts intersect, are crucial for understanding the dynamics of knowledge production and academic life. Tsing’s framework challenges the idea of frictionless progress, emphasizing how these encounters spark movements, reactions, and emotions that are key to understanding shifts in research cultures. This approach allows us to track the multiple matters of concern (Latour, 2004) that have emerged over time, revealing how the issues now subsumed under research integrity were gradually assembled. We cannot, therefore, speak of a single problem but of many context-specific frictions.

Frictions, then, are not mere conflicts but learning sites where power dynamics and cultural framings emerge, shaping how research is conducted and how academia functions. By reflecting on the ‘scientific self’ shaped in these narratives, we can also draw on Goffman (1959) to highlight that identity and self-worth are continuously developed in relation to others in a community. Through socialization, individuals learn the norms and values of the scientific culture of which they are part. Thus, the question of who constitutes the community that decides what is problematic in knowledge production—and how to respond to transgressions—becomes central to our analysis.

## Methodological Approach

### Nature and Science as Hybrid Spaces

As scientific journals, *Nature* and *Science* are spaces where researchers speak with the scientific community. Their scientific audiences, however, are not limited to a single disciplinary background but encompass a global, multidisciplinary readership. STS research on *Nature* and *Science* as epistemic spheres has focused, for instance, on the power of these top journals to set epistemic standards about what counts as evidence and who can contribute to knowledge production (Bonneuil et al., 2014), on how articles may represent the starting point for controversies about the contextualization of ethical engagement in knowledge production (Benjamin, 2016) or about the role of publications in such journals in negotiations of ownerships of knowledge (Swanson, 2007).

*Nature* and *Science* do not limit their actions to facilitating scholarly discourse, but also publish lengthy editorials, news sections, or letters (Ding et al., 2018) in which they also *reflect on the functioning* of the scientific community. There, they voice critique of structural imbalances in science and comment on science policies, global developments and science-society relationships. STS research drawing on this aspect illustrates, for example, the dominant position such prominent journals have for influencing the global perception of national research initiatives (Fukushima, 2016) or articles using quotes from old volumes as witnesses of a bygone scientific era to illustrate past worries and dynamics (Sapir, 2017). Kjaergaard’s (2011) article comparing the coverage of the misconduct case around Hwang Woo-Suk in *Nature* and *Science* is another example. The author shows how the journals’ positioning and the narratives they take up relate to the journals’ stakes in the case and broader geopolitical dynamics.

Furthermore, *Nature* and *Science* can also be understood as speaking for the scientific community in broader societal discourses. Their readerships are not limited to scientists (Krause, 2017); their inputs are also widely recognized in the policy realm. Here, STS researchers have, for instance, highlighted the close interaction of these two journals with news-media (Caldwell, 2017; Shea, 2001). Besides, reflections from their editors expressing concerns about systemic challenges in science are used to illustrate worries about the scientific community (Fochler et al., 2016).

Their particular position of being not only long-term witnesses but also architects of the discourse at stake makes the journals an ideal space to study shifts in why, for whom, where, when, and how research integrity should be a matter of concern. In that sense, they are also ideal spaces for observing the co-productive relationship between science and society (Felt & Davies, 2020). Authors and editors appropriate prevailing discourses in specific ways or by ‘selective[ly] retailoring them to suit new needs’ (Jasanoff, 2004, p. 41). Analysing the articles addressing issues of research integrity thus offers a window to understanding facets of the co-production of research and wider research environments.

### Data Collected

The material corpus for our research consisted of articles from 1980 to 2019. We chose 1980 as a starting point as around this moment several major cases of scientific misconduct were highly present and widely discussed in the scientific community and beyond. Our endpoint in 2019 was just before the Covid-19 crisis came into the picture, avoiding possible abrupt changes occurring as a result of Covid. In a first step, we worked from the journals’ webpages to identify a broad set of potentially relevant articles using the keywords ‘fraud’, ‘misconduct’, ‘dishonesty’, ‘falsification/falsify’, ‘plagiarism/plagiarize’ and ‘whistle-blower’. We also included linked articles and articles in special issues (e.g., on reproducibility crisis). Through this approach, we gathered 1192 articles from *Nature* and 1583 articles from *Science*. This set contained many different types of texts: editorials, research articles, opinion pieces, letters, and comments. As we were interested in how these journals would make sense of problems in research integrity, and thus construct its narratives, for our final corpus we only included texts that were at least half a page long. In total, we analysed 447 articles from *Nature* and 451 from *Science*.

### Narratives and Their Choreographies

Our analysis of the articles followed a narrative approach (Czarniawska, 2004). We understand the narratives in the two journals as culturally anchored stories that aim at giving meaningful accounts of the issues at stake and reflect on potential interventions. Investigating these narratives allows us to understand how authors make sense of science as legitimate practice and culture. Moreover, it allows us to follow how they map out the ‘geographies of responsibilities’ (Akrich, 1992; Sigl et al., 2020) at stake in the debates. The notion of geography here refers to ‘a multidimensional field, encompassing spatial (where effects manifest), temporal (when consequences unfold), ethical (who is affected and who is positioned to care or act), and affective (how responsibility is felt, distributed, or disavowed) dimensions’ (Felt, 2025b). It supports our attention to how responsibility is scripted, resisted, redefined, displaced, or fragmented across the gradually extending set of actors, institutions and infrastructures that constitute contemporary academic research environments. Following such narratives over time, allows us to grasp when, where, why, and by whom concerns are raised and who is called for solving the issue at stake. These narratives are, therefore, always diagnostic, telling us about the perception of the health of contemporary research systems (Penkler et al., 2015).

Narratives allow communities to share values and collectively make sense of practices. They can be understood as stabilizing elements that help define and reproduce research cultures. Investigating narratives thus allows us to capture how certain collective imaginations become stabilized or get questioned and replaced. We pay attention to the different forms in which we encounter narratives, which can range from ‘assessments, reconfigurations of past developments, [and] future-oriented accounts voicing promises’ to the identification of ‘potential threats and moral reflections of what is good science and innovation and how a good researcher should be’ (Felt, 2017, p. 56). Studying narratives can thus be understood as vital for ‘understand[ing] change beyond formal structural shifts’ (Felt, 2017, p. 55) and allow us to engage with gradually changing understandings of the imaginary of the scientific self on different levels.

Narratives order developments in space and time. They are ideal for examining transformations of these developments and enabling us to inquire about the changing conceptualizations of the scientific self in relation to irritations about transgressions of good scientific practice. While the corpus of articles shows a broad range of matters of concern identified across time when it comes to the self-governing capacity of science, our analysis identified five narrative strands that were elaborated and mobilized (see Table 1). These narrative strands change over time, grow in importance, or shift to the background and get entangled in ever-changing ways, sometimes forming discourse coalitions (Hajer, 2009) and sometimes creating frictions. These different strands are then woven together in situated ways, making some narratives more central while others must align with them to form a whole. In our analysis, we will describe this bringing together of narrative strands—each with its own actors, arguments, and temporal rhythms—as different narrative choreographies, i.e., as different entanglements of narrative strands, which communicate a specific form of situated coherence when it comes to capturing the situation of research integrity. As we will show, strands emerge, gain prominence, or fade; they interact—reinforcing, contradicting, or reframing each other. Speaking of choreographies, draws our attention to coordination, frictions and contrast, temporal dynamics, and positioning in the research landscape. These shifting choreographies then also point to the emergence of specific assemblages of the scientific self and their related responsibilities.

**Table 1.** Five Narrative Strands.

Narrative strand	Focus
Researchers and their community	Addresses the figure of the researcher and researchers’ roles and status in the community and beyond; reflects norms, values, and understandings of duty that would/should guide research practices.
Epistemic self-regulation processes and practices	Engages with questions of how knowledge gets critically scrutinized, validated, and revised; discusses organized scepticism, and how good evidence/good data is defined and assessed.
Institutional actors	Draws attention to the different institutional actors and their roles in research, such as funding agencies, institutions where research is done (e.g., universities or industrial labs), journals or bodies watching over research integrity.
Research environment	Discusses the major changes in the ways research is organized and assessed, how careers are practiced, and how all this impacts research integrity culture.
Relation to wider society	Reflects on the place of science in society, on the trust relationships essential to science and how issues around research integrity could have negative impacts if not addressed accordingly.

The five narrative strands we identified 1) address the figure of the researcher and respective communities, 2) cover epistemic self-regulation processes and practices that are in place at a specific moment in time, 3) reflect on institutional actors that matter to the way research works, 4) open debates on the often quite fundamental changes in the research environments and 5) touch on the changing relations of science and society.

## Findings: Major Shifts in Response to Transgressions of Good Scientific Practice

By analysing how these five narrative strands come together in complex narrative choreographies, we structure the timespan under investigation into three different phases: up to the late 1980s, from the late 1980s to the early 2000s, and from the early 2000s onward. Such periodizations are always difficult to establish, because the temporal boundaries we draw are fuzzy and porous, as the different elements of each phase continue their lives in the next phases, albeit in different ways. All five narrative strands are co-present across the entire timespan of the study, yet their choreography and the role of each strand shifted considerably over time. As with any periodization, ours is shaped by the empirical events and sources we consider most indicative of the transformations under analysis—thus capturing a broader vision of debates around issues of transgression. Other scholars, such as Montgomery and Oliver (2009), arrive at a somewhat different temporal mapping in their study of the development and dissemination of ethical guidelines from 1975 to the present. Drawing on institutional change at the organizational-field level in the US context, their work examines the co-evolution of the structure of the scientific research field and its underlying institutional logic—an approach that foregrounds different actors and processes, and thus produces a different, yet complementary, historical framing.

## Phase 1 (Until the Late 1980s): Is Scientific Fraud a Major Problem for Science? Imagining a Family Business That Cares for *Correcting*

Our analysis starts in the 1980s, a moment in time that seemed to witness an increasing number of reports concerning research practice, mainly in the form of stories on fabricated data and plagiarism. This early phase is of special interest because we can observe quite intense negotiations around the question of whether transgressions of good scientific practice are becoming more frequent and whether the scientific community has the capacity to address this problem internally. ‘The truth is’, one article argued, ‘that no one knows whether fraud is rare or not or whether it will inevitably be found out’ [*Science*, 1983^3^].

Among the choreographies of transgression stories in the 1980s, the narrative strand gravitating around the role of the individual researcher and their community is at the core of articles, albeit with considerable variations. Articles typically focused on (alleged) malpractice instances, highlighting individual scientists’ actions and moral dispositions. Some, particularly in *Science*, read like detective stories, gradually revealing the ‘rotten apple’ responsible for violating scientific norms. These accounts invited readers to follow the detectives’ forensic work as they uncovered fraud, often presenting complex, messy situations involving the falsification of data to ‘obtain a result nearer to the expected figure’, a ‘notebook of forged data’, sloppy research practices, or a failure to respond to community criticism—issues that were either deliberately hidden or obscured by a strong bias to see things in a particular light [e.g., *Science*, 1981^4^].

Researchers who have violated accepted norms are often portrayed as individuals of questionable character, failing to adhere to the ethical principles central to research and engaging in self-interested or morally dubious behaviour. Transgressions are typically attributed to a single individual, with younger scholars disproportionately represented in such cases. Their failings are framed as a disregard for the ideal of science as a community bound by shared values. In this view, adherence to a common work ethos is positioned as fundamental to the smooth functioning of the scientific system, reflecting a Mertonian (1973 [1968]) understanding of the scientific ethos. Researchers’ lack of moral integrity was perceived as undermining the collective effort to produce reliable knowledge and eroding trust within the scientific community— ‘scientists cannot trust each other’ [*Nature*, 1981^5^].

During this period, the issue of trust is primarily framed in terms of the internal dynamics of the scientific community, with public trust in science not yet a central concern. Public awareness of scientific transgressions is largely dismissed as irrelevant. In this context, societal actors primarily appear as political figures, such as members of the U.S. Congress during the 1981 hearings on scientific fraud convened by Al Gore. However, these hearings did not lead to significant consequences.

While those who are identified as fraudsters fall into a clear-cut moral category, the figure of the whistle-blower, i.e., the person denouncing a situation of misconduct, is met with deep ambivalence, even though they frequently play an essential role in the ‘detective stories’. Indeed, as outlined in the early 1980s, the then-recent cases of transgressions show that well beyond usual practices of control and replication, ‘other means have predominated’ in the discovery of fraud. This included, for example, ‘detective work of young lab assistants or young scientific rivals who have extra-experimental evidence of cheating, who have some independent reason for suspicion’ [*Science*, 1981^6^]. However, while this is mentioned as an important part of self-correction, we encounter several cases where whistle-blowers face difficult situations, fearing they might lose their jobs, being suspected of acting out of personal interest or experiencing social exclusion. Thus, they were perceived as betraying the community and tarnishing its positive image by revealing ‘family secrets’ to those outside the fold.

Being part of the scientific family comes with high expectations and the assumption that researchers act as members of a value community. Scientists who are found guilty of scientific fraud are, as per the narrative, ‘as a matter of routine [expelled] from the scientific community’ [*Nature*, 1981^7^]. The judges of whether someone transgressed the boundaries of acceptable research practices are part of the direct research environment, thus the department or laboratory members where the research in question was conducted. They were supposed to act as agents to assure the quality of research. Thus, ‘the objective should be to make sure that laboratory communities remain as rigorous as they should be in their internal assessment of all their members’ [*Nature*, 1981^8^].

Scepticism, organized through reproducing and building on the results of other scientists as well as a critical engagement with peers, is deemed sufficient to detect and sort out wrong claims over time. The same holds true for both unavoidable ‘honest mistakes’ as well as for deliberately misrepresented results. If an experiment ‘appears to have significance it will be repeated and retested in numerous ways’ [*Science*, 1981^9^] is a frequent line of argumentation. Also, the peer-review process in journals is seen as an essential means to critically scrutinize results, even though the limitations are often pointed out. While we can trace the belief that fraud ‘will probably be exposed by the scientific process in the long run’, it is also admitted that ‘this is not necessarily true of all fraud and certainly does not apply to the short run’ [*Nature*, 1983^10^].

The assurances that science will correct itself by filtering out wrong claims and expelling individuals who do not follow the moral code enable the belief that transgressions do not significantly harm science. For example, in reports on the congressional hearing mentioned above, officials from the NIH explicitly ‘den[ied] that fraud is a significant problem overall’, stressing the need to be cautious about making quick judgments on whether something constitutes a serious transgression or not [*Science*, 1981^11^].

Placing scientists, their communities, and practices of epistemic self-correction at centre stage for responding to transgressions also comes with a reluctance to attribute too important a role to institutions. Doubts are raised about whether institutions, such as universities or funding agencies, are sufficiently prepared to execute sanctions for scientific fraud formally. They were frequently described as reacting too hesitantly in the context of transgressions, tending to ‘look away’, and ignoring potentially problematic scientists. This is particularly true when researchers managed to become highly visible and thus somewhat achieved ‘immunity from scrutiny’ [*Science*, 1981^12^]. Universities are accused of wanting to protect their reputations and, hence, not thoroughly investigating accusations of scientific misconduct.

While institutional actors are seen as less significant for governing research practices than the scientific value collective, we can see the first instances when the changing research environment was described as a potential cause for rising transgressions. Several articles point to the ‘significant pressure to publish … data as fast as possible so as to obtain priority’ [*Science*, 1980^13^] and secure grants and stable positions, which may tempt researchers to cut corners. Yet, these initial observations, suggesting that the issue might not be merely ‘a matter of rotten apples in the barrel’ but rather something intrinsic to ‘the barrel itself’ [*Nature*, 1983^14^], are frequently dismissed as mere rationalizations, diverting attention from the individual’s failure to uphold the core values traditionally seen as guiding science [e.g., *Science*, 1983^15^].

**Figure 1.**
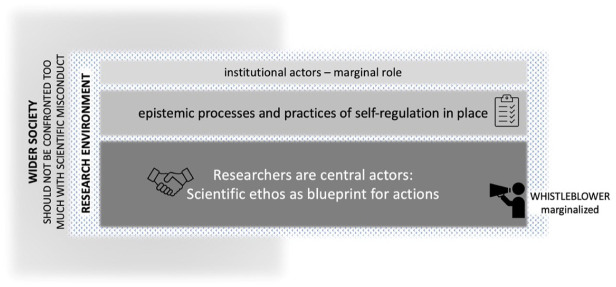
Choreography of the five narrative strands in phase 1—The family-business model.

As such, in 1980s *Nature* and *Science* articles, science is described as able to govern itself, handling transgressions as a ‘family business’ (Figure 1). Scientists desire minimal interference from societal or institutional actors in overseeing or regulating their practices. Responsibility for research is described as lying in the relationships among peers, colleagues, and supervisors, who were supposed to review the produced knowledge critically. Research is presented as a deeply cultural process, where scientists are expected to act according to core values. Governing scientific misconduct often boils down to expelling fraudsters who do not adhere to these ideals. Only those closely involved in the research process can judge and regulate the few cases where individual ‘rotten apples’ misbehaved and engaged in fraud. Mertonian ideals about science, picturing scientists as a community of values following implicit norms are prevalent and allow to cherish the idea of automatic self-correction of results as science advances. Institutional actors are not deemed necessary for this ‘purely scientific’ process among scientists.

## Phase 2 (Late 1980s to Early 2000s): Scientific Misconduct is a Problem! Including Institutional Actors in a ‘Scientific Self’ That *Regulates*

In the second phase, which largely spans the 1990s, debates about transgressions of good scientific practice increasingly invited wider public debate. Several Congressional hearings were held in the U.S. to investigate the potential waste of taxpayers’ money, and newspapers increasingly reported accusations of scientific misconduct. Societal scrutiny of the questionable behaviour of individual scientists was seen as unsettling, not only for its potential to erode public trust in research but also due to a palpable fear of external societal oversight. Institutions were publicly warned that:

Fraud would eventually come out—it always does—and that newspaper accounts of a cover-up would do far more damage to the public trust than a clear statement about a single [person] … who thought he could bend the rules and get away with it. [*Nature*, 1994^16^]

Indeed, society started to speak back to science (Nowotny et al., 2001) in these years, and this discursive strand now occupied a more central position in the overall narrative choreography. The period when science operated as a largely unquestioned and successful enterprise and could keep its intricate processes hidden from public scrutiny seemed to have come to an end.

The self-correction argument so strongly advocated for in the first period started to lose its power. Calls were now regularly made to ‘explicitly acknowledge that science’s self-correcting mechanisms are insufficient to guard against dishonest research’ [*Science*, 1992^17^]. The epistemic processes and practices of self-correction, filtering out wrong claims over time and expelling individuals—the ‘in-the-end-good-science-wins’ argument—were described as important but too fragile in their nature, and clearer articulations of formal control and oversight would be needed. Such formal procedures of investigation and the sanctioning of transgressions would, however, need considerable investments of time and resources, which only institutional actors were seen in the position to care for. This explains why institutional actors and their responsibilities moved to the centre stage of the debate. However, in this context, it was important that institutional actors use scientists to study those cases, as there was a strong belief that ‘scientists, not lawyers or accountants, should be the first to decide whether a researcher had behaved inproperly’ and that shifting such decisions to courts creates a risk that ‘all of the groundwork painstakingly covered to deal with these issues will be out the window’. [*Science*, 1990^18^].

Once diverse institutional actors were expected to take on responsibilities, there were more explicit attempts to define scientific misconduct and establish rules and regulations. However, these collective reflections went into rather different directions. Some showed concerns that ‘public attention tends to focus on dramatic instances of misconduct—the occasional cases of fabrication, falsification, and plagiarism that all agree violate the ethical norms of science’ [*Science*, 1994^19^]. This might explain why high-level norms were made explicit in universities’ and funding agencies’ newly written codes of conduct—to assure societal actors that the issue of transgressions was taken seriously on an institutional level. However, we also found voices stressing that more attention was needed to ‘those behaviours that do not rise to the level of misconduct but nevertheless violate values held in common by the scientific community’ [*Science*, 1994]. Examples mentioned were the ‘allocation of credit’ for contributions to research, ‘the treatment of research data’ which often required considerable time and care that remained largely invisible in reward systems, and ‘mentorship responsibilities’ [*Science*, 1994]. By opening up the debate to a much wider array of issues came disagreement about whether we would only see the tip of the iceberg of research transgressions. Indeed, the number of registered cases remained low compared to the total number of publications or cases reported in universities. Still, there were articles reporting on surveys finding a considerable number of researchers witnessing ‘serious breaches of research ethical guidelines’ [*Nature*, 1999^20^]. Despite all the debates around definitions and reliable numbers of misconduct incidents, there was agreement that scientific misconduct was an issue that needed to be taken seriously.

It is thus beyond doubt that institutional actors were seen as having ‘a responsibility to articulate standards for ethical conduct and to see that they are put into practice’ [*Science*, 1997^21^]. Concrete rules, investigative processes, and procedures for handling misconduct allegations thus become central elements in the discursive strand on institutions. We can also follow the introduction of offices for research integrity, which struggled in the first years to find their positions but gained legitimacy during this phase. While it is clear that the very meaning of the scientific self was extended by institutional actors, who has to investigated what, how, and when still required clarification. This was for example addressed, when stating ‘academic institutions that receive their research funds … investigate allegations of misconduct in the first instance, and then … report their findings to the funding agency’ [*Nature*, 1999^22^]. In parallel, institutions also considered how to ensure that novice researchers grew into science and were made aware of the newly implemented rules. Funding bodies, therefore, made it obligatory that ‘institutions receiving … grants had to provide instruction in research ethics to their young researchers’ [*Science*, 1995^23^].

However, these new social arrangements also raised other considerations: ‘The new procedures [for investigating scientific misconduct] are designed to ensure that cases of scientific misconduct are dealt with rapidly, and that both whistle-blowers and those innocently accused are protected’ [*Nature*, 1997^24^]. Introducing such processes aimed to ensure fair and accountable responses to accusations, addressing criticisms of arbitrariness in previous ad-hoc investigations. Yet, striking a balance between safeguarding all parties involved—and especially the accuser and the accused—remained a challenge. Reports continued to highlight ambivalence and outright hostility towards researchers who came forward with concerns about the improper behaviour of colleagues or supervisors. Whistle-blowers, often close colleagues or students, not only bore the emotional toll of initiating investigations but also played a pivotal role in the evolving landscape of research integrity within newly implemented university frameworks.

Another group of institutional actors that gained increasing prominence in the second phase was scientific journals. In the first phase, they present more as commentators on events related to research integrity and as facilitators of scientific exchange without much intervention (beyond peer review). However, here they began to explicitly reflect on potential roles, responsibilities, possibilities, and challenges. This repositioning was nicely captured in a statement in *Nature*, underlining that journals are

An important layer of defence, detecting error (more easily than fraud) before it entered the scientific record, providing a forum for disagreement (journals without correspondence columns were urged to provide them), and ensuring that notice be given of research found fraudulent. [*Nature*, 1989^25^]

The heightened attention must be viewed in the context of persistent complaints that journals have been reluctant to address research misconduct issues, too slow to retract problematic papers, and hesitant to engage in inquiries concerning research integrity. In response, journal efforts during these years concentrated on enforcing clear guidelines, particularly regarding authorship criteria [*Nature*, 1989^26^; *Science*, 1997^27^] or peer review practices [*Science*, 1995^28^]. In subsequent years, these efforts expanded to include standards for permissible manipulations of digital images in published articles [*Science*, 1994^29^].

In the initial phase, research environments were acknowledged as potential contributors to boundary transgressions, yet such systemic factors were simultaneously dismissed as unacceptable excuses for individual misconduct. Over time, however, the systemic dimensions and dominant values within which researchers operate have undergone such profound changes that articles in leading journals increasingly suggest causal links between these factors and violations of good scientific practice. References frequently highlighted the intensifying competitiveness of the field, the relentless pressure on researchers to publish prolifically, and the fact that ‘promotions, appointments and the award of research grants [depend more than ever before] on people’s publication records’ [*Nature*, 1994^30^]. This, in turn, leads researchers to ‘try to magnify their reputation by publishing more than they need (and have to say)’ and it explains

People’s eagerness to be co-authors of articles of whose content they are largely ignorant (and whose conclusions they are unable to defend), and … the inadequate and even misleading attributions of credit for earlier work now commonplace in the literature. [*Nature*, 1994]

**Figure 2.**
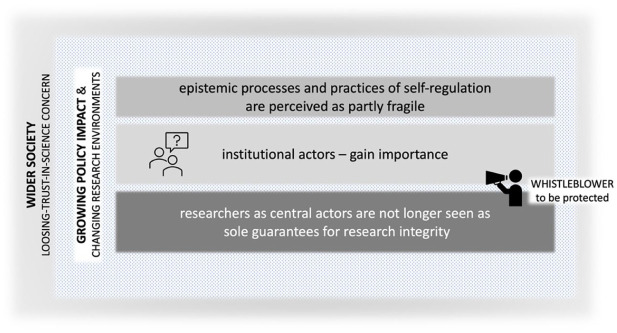
Choreography of the five narrative strands in phase 2—An institutionally extended self model.

Therefore, the second phase reflects a broader understanding of scientific misconduct and a redefined conception of the scientific self, now seen as an institutionally extended entity. Institutional actors increasingly played a pivotal role in shaping scientific practice, complementing the traditional Mertonian ideal, which is gradually viewed as insufficient for addressing misconduct. The fragility of self-correcting epistemic processes underscored the need for broader systemic regulation. This evolution mirrored shifts in the research ecosystem and the deepening entanglement of science with society, redistributing responsibility for research integrity across diverse institutional actors. Consequently, practices for ensuring integrity were becoming more formalized, marking a shift from self-correction to structured self-regulation (Figure 2).

## Phase 3 (From the Early 2000s Onwards): Confronting Research Integrity—Governance by a Multi-stakeholder Self That *Manages* Science

The third phase began in the early 2000s, marked by prominent international fraud cases that cast doubt on the sufficiency of established institutional self-regulation in addressing integrity issues. What raised concern was the scale of transgressions committed by respected scientists such as materials scientist Jan Hendrik Schön and stem-cell researcher Hwang Woo-Suk. Numerous articles in leading journals conducted quasi-autopsies of these cases, dissecting systemic failures and highlighting dysfunctions in research processes. These analyses exposed flaws and provoked substantial critiques of the research ecosystem’s prevailing social and institutional structures. Some articles depicted these fraud cases as ‘learning experience [that] will be a stepping stone for better execution and management of scientific research’ [*Nature*, 2006^31^].

Schön and Hwang’s cases clearly exposed the publication system’s fragility. *Nature* and *Science* came under particular pressure, fearing for their reputation, because they had published many papers that had to be retracted. In response, they ramped up regulatory efforts, building on previous initiatives by introducing stricter authorship and peer review guidelines. Notably, editorials took pride in the added review mechanisms, including using algorithmic tools to detect statistical errors, plagiarism, and image manipulation—marking a more proactive approach to preserving research integrity. ‘If you look at the number of mistakes we manage to correct before publication, I think it is worth it’ said the responsible ‘image detective’ at *Nature* in an interview, further stating: ‘It’s especially nice to get replies from authors who are really grateful when we spot errors. We are protecting the published record and their reputation’ [*Nature*, 2015^32^]. While journals had positioned themselves earlier as facilitating communication among scientists with minimal interference, they increasingly adopted more active, managerial roles, balancing oversight with support to uphold research integrity and guide researchers in maintaining standards.

One notable shift is the increased space journals provided for researchers to share perspectives through commentary pieces, letters, and reports on scientific misconduct, which are eagerly embraced by the scientific community. Beyond anecdotal accounts, researchers demonstrated a growing epistemic interest in studying misconduct, with articles reporting on interviews, discussion groups, and surveys that capture their perspectives and observations on breaches of good scientific practice [e.g., *Nature*, 2003^33^]. These studies highlighted that the spectrum of matters of concern is for researchers much wider than the proclaimed focus on outright misconduct. Increasingly, we observe how scientists ‘put science under the microscope’ [*Nature*, 2016^34^] and discussions zoom in on potentially worrying mundane research practices, such as the use of statistics, alterations in scientific illustration, the living conditions of animals, incoherent labelling of antibodies, unconscious biases, conflicts of interests, and the overall replicability of published results [e.g., *Nature*, 2001^35^; *Nature*, 2012^36^; *Science*, 2013^37^; *Science*, 2014^38^; *Nature*, 2015^39^; *Science*, 2016^40^; *Nature*, 2016^41^]. This increased self-reflection, which is also portrayed as a form of epistemic self-regulation, contributed to a greater use of the term ‘research integrity’ and we also saw the range of practices considered concerning to expand.

Discussions of research integrity also started to intersect with growing concerns about how contemporary research environments—often in reference to the neoliberal transformation of research (Felt, 2025a; Fochler et al., 2016)—affect the production of reliable knowledge. Competitiveness was increasingly portrayed as central to the research landscape and publications were framed as commodities through which individuals (and their institutions) compete in the global research system. However, these dynamics were often criticized for fostering conditions that may lead to transgressions, careless practices, or the erosion of trustworthy knowledge. The relentless pressure to publish, precarious career paths, limited time for meaningful mentorship, toxic lab cultures, high mobility, and other environmental factors were seen as creating vulnerabilities that encourage transgressions while leaving insufficient space to reflect on how research is done. Studies were reported to show that researchers ‘are more likely to behave unethically if they believe their managers are treating them unfairly’ [*Nature*, 2007^42^]. This led to a paradoxical situation: ‘many of the risk factors for misconduct also seem to be what makes for good science’ [*Nature*, 2007].

Furthermore, increased attention was given to science-society relationships which were seen as endangered through these developments: ‘Nothing will erode public trust more than a creeping awareness that scientists are unable to live up to the standards that they have set for themselves’ [*Nature*, 2012^43^]. Throughout the third phase, we repeatedly read about the importance of protecting the reliability of research to legitimize the role of science and ensure its contribution to society. Different from the second phase, when the fear of societal control was palpable, it was now about having a healthy relationship with society. In particular, high-profile cases, which attracted the attention of journalists, were seen as worrisome and as potentially harmful for wider trust relationships.

Faced with a broadening spectrum of concerns—including not only scientific fraud and misconduct but also more routine questionable practices—we observed the enforcement of a patchwork of responses. As noted [*Science*, 2015^44^], ‘ensuring that the integrity of science is protected is [seen as] the responsibility of many stakeholders.’ Around each issue, actors assembled to negotiate their capacities and responsibilities in addressing problematic behaviour. For long-standing concerns, institutional processes often stabilized, sometimes becoming so entrenched they were treated as unquestionable gold standards. Countries lacking institutionalized mechanisms for investigating misconduct were frequently criticized, with their responses compared against these established norms. In contrast, responses to newly emerging concerns remained subject to negotiation. Yet, whether addressing old or new issues, most contemporary responses shared common strategies: formalizing (e.g., international misconduct rules, guidelines for animal research, conflict of interest policies), standardizing (e.g., harmonizing global investigative procedures, standardizing laboratory techniques and data-sharing formats), and controlling (e.g., algorithmic checks for statistics and plagiarism, automated experimental protocols, video documentation of processes). Collectively, we describe these as managerial approaches to research integrity.

Efforts to manage research integrity were underscored by a strong emphasis on transparency. Advocating openness aligned well with managerial approaches of auditing and accountability. Making ‘data, samples, methods, and reagents’ accessible [*Science*, 2016^45^] was seen as enhancing reliability—not only by enabling scrutiny but also by fostering a sense of responsibility among researchers. Universities, too, were urged to adopt transparency, especially in misconduct investigations.

Once an investigation is complete, the institution should be as transparent as it can about what happened. Especially when public funds are involved … the taxpayer has a right to know what happened when papers are retracted—even if the faculty member in question is eventually exonerated. [*Nature*, 2011^46^]

In this context, the public arena was reframed: not merely as a potential threat to trust in science but as a space where transparency can help restore trust. The rise of the internet and digital tools revolutionized how data, critique, and ideas circulate. Initiatives like Open Access increased visibility into research processes, while platforms such as PubPeer and Retraction Watch ‘democratize’ critique, allowing broader participation in identifying errors or voicing concerns. These platforms also redefined whistleblowing as a systemic responsibility, with anonymity fostering a culture of accountability. This aligns with the evolving notion that ‘peer review continues long after a paper is published’, becoming part of the ongoing scientific record [*Nature*, 2011^47^]. Connectivity was also celebrated for its practicality, with one editorial [*Nature*, 2015^48^] suggesting that researchers struggling to reproduce results could find help ‘just a phone call away’.

In this phase, researchers themselves are portrayed as key managerial actors. Innovators who create online infrastructures of sharing and exchange are highlighted as entrepreneurs shaping reliable practices. Additionally, the growing spectrum of concerns—extending into everyday research practices—placed new responsibilities on researchers. They were expected to manage their work meticulously by providing data management plans (*Science*, 2016^49^], addressing biases (*Nature*, 2015^50^], and improving lab-management habits (*Nature*, 2016^51^]. As central stakeholders, researchers were tasked with formalizing, standardizing, and controlling their own processes to ensure high-quality science.

This managerial turn has not gone without challenge. Editorials and commentaries increasingly call for rethinking and reorganizing science, proposing alternatives that move beyond managerialism. These fragmented visions aim to address present challenges while fostering a forward-looking ethos. By the end of this phase, a shared narrative emerged, emphasizing the need for scientists to embrace responsibility and transparency, cultivating a collective ethos to guide the future of research.

**Figure 3.**
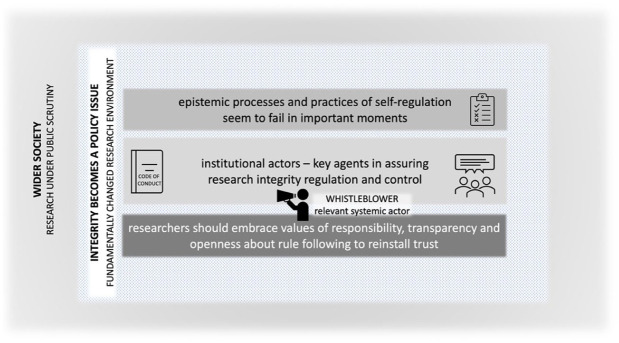
Choreography of the five narrative strands in phase 3—A managerial multi-stakeholder-self model.

Therefore, the third phase is characterized by a model of the multi-stakeholder managerial self (Figure 3). Faced with the complex challenges to research integrity and the recognition that existing self-correction and regulation processes often fail at critical moments, new normative procedures for control were proposed. These included the introduction of standardization mechanisms alongside a redefinition and formalization of roles. However, stakeholders are not seen as acting in isolation; rather, they are understood to be deeply interconnected. Only networked approaches, where the responsibilities of each member of the scientific self were adapted to the specific situation, are considered viable. And it is essential to mention that legal procedures, even though there were more reports during this phase, are still being seen as coming after the fact at moments where the multi-stakeholder managerial self made the basic decision on whether something was to be regarded as transgression of research integrity.

## Discussion and Conclusions

This article traces four decades of discussions on scientific misconduct in *Nature* and *Science*. By questioning the myth of science’s self-corrective capacity and examining how the concept of the ‘scientific self’ has evolved over time, we gained valuable insights on two levels. First, we could see how transformations in the research system and the handling of transgressions of good practice are closely entangled, and how this shapes the ways distribution of responsibility is envisaged. Second, we saw the gradual shifts in the complex relationships between science and society and how this mattered in the way transgressions were being addressed.

Identifying five narrative strands across this 40-year period, we distinguished three phases. In phase 1 (until the late 1980s), the focus was on researchers and a relatively insular vision of the scientific community, closely aligned with the Mertonian ideal (Merton, 1973 [1968]). Researchers were expected to be guided by this ethos, with science’s self-corrective capacity framed as an internal process akin to a *family business*. A sharp boundary was drawn between science and society and between the scientific community and institutional actors supporting science. The prevailing view was that only scientists can judge what constitutes adequate practice. A wider society only appears at the margins, mainly constructed as profiting from scientific insights and not needing to know what happens behind the scenes.

As the conditions for research and its relationship with society evolved, phase 2 (from late 1980s to early 2000s) witnessed the emergence of a broader *institutionally extended self model*, coopting institutional actors into the scientific community to address misconduct. This phase was marked by experimentation and uncertainty. We witnessed not only an expansion in the geography of responsibility, involving more actors than before, but also the considerable expansion of the definition of transgression, going well beyond classical fraud (Resnik, 2003). Public trust in science, more essential than ever before, was seen as in jeopardy. This prompted institutional actors—particularly universities, funding agencies, and research integrity offices—to take on more central roles, and the scientific community had to extend their understanding of the scientific self. This inclusion of institutional actors and their efforts to introduce new rules and processes were framed as a means to maintain science’s ability to self-regulate.

By phase 3 (from early 2000s onwards), the role of institutional actors was firmly established, leading to more formalized control mechanisms. Indeed, as research integrity issues grew in complexity, a networked, multi-stakeholder approach was increasingly seen as necessary, with scientists, universities, funding agencies, journals, and research integrity offices all playing a role in safeguarding the trustworthiness of scientific knowledge. While involving these actors in ensuring research integrity is crucial, an overreliance on control mechanisms risks transforming reflections on good practice and research integrity into a ‘bureaucracy of virtues’ (Felt, 2017), i.e., a codified set of values and procedures, leading to a growing focus on formal compliance rather than meaningful accountability. Furthermore, increasingly questions appeared regarding whether the transformation of research is not also a potential source for fostering transgressions. Therefore, we would argue that the ‘boundary between healthy skepticism and manufactured doubt’ was no longer a ‘simple matter of distinguishing between valid laboratory and publishing protocols on one side and political, economic, or personal interests on the other’ (Roberts et al., 2020). Research and the transformation of both science and society are deeply entangled with each other and therefore situated, and careful answers must be found to assure a culture of integrity. Indeed, the *managerial, multi-stakeholder model* also clearly showed its limitations, especially as contemporary research incentives encouraged shortcuts and strained collaborative relationships. These developments were closely scrutinized, with growing concern over the erosion of public trust in science.

Across all three phases, the role of whistleblowing evolved significantly. Initially marginalized and often viewed with disdain, whistleblowers gradually came to be recognized as figures deserving protection from attacks within the scientific community. By the end of the third phase, they had gained an integral role in upholding research integrity.

Through a longitudinal analysis of debates on research integrity, we saw how misconduct issues offered a lens for understanding the co-production of social and knowledge orders within research. Our analysis highlights the value of studying ‘frictions’, in the sense proposed by Tsing (2005, p. 1), i.e., as opportunities to examine the ‘grip of worldly encounters’ researchers have with the changing realities they must navigate. By focusing on these frictions, we become attuned to the power dynamics at play across the three phases, recognizing how much transgressions of good practice are shaping and being shaped by how research is transformed.

We witnessed intense exercises of demarcation within science, as well as between science and society (Gieryn, 1999). Yet at the same time we observed how the self that should be the agent of self-governance expanded and changed. Gradually, different institutional players were integrated into the models of the scientific self that should ensure good scientific practice, challenging earlier demarcations. This was due to two important transformations taking place during these 40 years. On one hand, we observed a clear shift in the dynamic but fragile relationship between science and society. Science could no longer be narrated as a detached, objective pursuit, but rather as an interactive and socially embedded practice that should increasingly contribute to identifying societal challenges and finding solutions. In a world marked by uncertainty and complexity, science was expected to be more responsive, inclusive, and transparent to gain trust in complex problem-solving tasks.

Yet, simultaneously, the research system was transformed under a neoliberal logic. These changes introduced market-like mentalities, more competitive behaviour, ever-shorter time frames for research, and sometimes subordination to economic, political, and institutional imperatives. While these shifts brought about efficiencies and focused attention on applying research to societal problems, they also increased the readiness to transgress boundaries (Felt, 2025a). This was reflected in the shift from correcting a few mistakes to regulating good practice to a more managerial approach to addressing transgressions.

Finally, it is illuminating to consider the observed transformation of the scientific self through the lens of Abbott’s (1988) work on professions. Abbott portrays professions as engaged in a continual struggle for jurisdiction—the right to define and control their own work—in a shifting landscape of relations with other professions. He identifies three primary arenas in which such jurisdiction is asserted: the workplace, the legal system, and public opinion, each shaping professional identity in distinct ways. In our analysis, we see how science has long sought to defend its jurisdiction over the definition and policing of misconduct, preserving internal authority for as long as possible. Yet this defense is evolving. The traditional reliance on internal peer regulation has gradually—if unevenly—given way to a shared regulatory space involving funders and other institutional actors. This shift is triggered by forces such as the projectification of research, the pressure of publication metrics, and the imperative of cultivating public trust to sustain independence (Felt, 2025a). What emerges is a negotiation between cultural legitimacy and external accountability—an ongoing balancing act between the ideal of the autonomous ‘scientific self’ and the demands of new governance structures. Notably, there remains a strong impulse to maintain a degree of separation from the legal system, ensuring that decisions on research integrity remain, as far and as long as possible, within the domain of the research community writ large. We started this article with a recent statement [*Nature*, 2024] stressing that ‘[r]etractions are part of science, but misconduct isn’t’ and underlining that ‘journals, funders and institutions that employ researchers … all can learn lessons from misconduct cases’. This is much in line with a noticeable shift in recent years, a shift towards emphasizing the importance of fostering a sense of belonging—not just to a community of practice, but to a broader community that shares common values and norms. This shift signals a growing commitment to collective stewardship of research integrity, with the aim of strengthening ethical practices in the future. While a strong managerial approach to research integrity still prevails, we are beginning to see the emergence of awareness regarding the limitations of this model. In response, there are early indications that more holistic, care-oriented approaches are being considered, aimed at strengthening ethical *practices* for the future. While managerial, compliance-driven approaches to integrity remain dominant, there is increasing recognition of their limits. In response, early signs point to the emergence of more holistic, care-oriented approaches—approaches that prioritize relational trust, mutual support, and ongoing dialogue over box-ticking and procedural audits.

Care is a practice that is relational, context-sensitive, and attentive to the needs of others. In research settings, this might mean establishing mentoring structures where senior scholars not only convey formal ethical rules but also model reflexive decision-making, openly discuss moral grey zones, and share responsibility for navigating ethical dilemmas as they arise. Rather than viewing integrity as a one-off formality—rules signed upon entering a field—integrity becomes a dynamic, continuous process, cultivated through everyday interactions, shared reflection, and mutual accountability (e.g. Royal Society & UK Research Integrity Office, 2018). Developing a shared understanding of what constitutes quality research and how to address ethical violations calls for collective reflection and mutual responsibility. In this sense, the focus is shifting from compliance-based models to those that encourage a deeper, more sustained commitment to ethical practice, one that acknowledges the complexities of research and the importance of maintaining trust within both the scientific community and society at large.

## Notes

1. Joyce, C. (1986) Deception beyond measure. *Nature*, *324*(6093), 181.

2. *Nature*. (2024). Editorial: Retractions are part of science, but misconduct isn’t. *Nature*, *628*(8009), 689.

3. Angell, M. (1983). Fraud in science. *Science*, *219*(4591), 1417–1418.

4. Wade, N. (1981). A diversion of the quest for truth. *Science*, *211*(4486), 1022–1025.

5. (1981). Responsibility for trust in research. *Nature*, *289*, 211–212.

6. Broad, W. (1981). Fraud and the structure of science. *Science*, *212*(4491), 137–141.

7. (1981). Who, not congress, should police fraud? *Nature*, *290*(5896), 433–444.

8. (1981). Responsibility for trust in research. *Nature*, *289*, 211–212.

9. Broad, W. (1981). Congress told fraud issue “exaggerated”. *Science*, *212*(4493), 421.

10. Angell, M. (1983). Fraud in science. *Science*, *219*(4591), 1417–1418.

11. Broad, W. (1981). Congress told fraud issue “exaggerated”. *Science*, *212*(4493), 421.

12. Broad, W. (1981). Fraud and the structure of science. *Science*, *212*(4491), 137–141.

13. Broad, W. (1980). Imbroglio at Yale (I): Emergence of a fraud. *Science*, *210*(4465), 38–41.

14. David, P. (1983). The system defends itself. *Nature*, *303*(5916), 369.

15. Culliton, B. (1983). Coping with fraud: the Darsee case. *Science*, *220*(4592), 31–35.

16. (1994). How to lose confidence. *Nature*, *368*(6473), 674.

17. Hamilton, D. (1992). A shaky consensus on misconduct. *Science*, *256*(5057), 604.

18. Palca, J. (1990). Old law puts a new wrinkle in fraud probes. *Science*, *247*(1990), 802.

19. Alberts, B., & Shine, K. (1994). Scientists and the integrity of research. *Science*, *266*(5191), 1660.

20. Abbott, A. (1999). Science comes to terms with the lessons of fraud. *Nature*, *398*(6722), 13–17.

21. Gunsalus, C. (1997). Ethics: Sending out the message. *Science*, *276*(5311), 335.

22. Abbott, A. (1999). Science comes to terms with the lessons of fraud. *Nature*, *398*(6722), 13–17.

23. Taubes, G. (1995). Indiana: Wrong answers – but no right ones. *Science*, *268*(5218), 1707.

24. Abbott, A. (1997). Germany tightens grip on misconduct. *Nature*, *390*(6659), 430.

25. Anderson, A. (1989). Scientific misconduct still an unknown. *Nature*, *340*(6228), 3.

26. Palca, J. (1989). Disputed paper takes centre stage in Congress. *Nature*, *339*(6220), 83.

27. Gunsalus, C. (1997). Ethics: Sending out the message. *Science*, *276*(5311), 335.

28. Marshall, E. (1995). Peer review: Written and unwritten rules. *Science*, *270*(5244), 1913.

29. Anderson, C. (1994). Easy-to-alter digital images raise fears of tampering. *Science*, *263*(5145), 317.

30. (1994). What to do about scientific misconduct. *Nature*, *369*(6478), 261–262.

31. Cyranoski, D. (2006). Verdict: Hwang’s human stem cells were all fakes. *Nature*, *439*(7073), 122–123.

32. Van Noorden, R. (2015). The image detective who roots out manuscript flaws. *Nature News*. https://doi.org/10.1038/nature.2015.17749

33. Abbott, A., & Graf, P. (2003). Survey reveals mixed feelings over scientific misconduct. *Nature*, *424*(6945), 117.

34. Lewandowsky, S., & Bishop, D. (2016). Don’t let transparency damage science. *Nature*, *529*(7587), 459–461.

35. Richardson, M. K., & Keuck, G. (2001). A question of intent: When is a ‘schematic’ illustration a fraud? *Nature*, *410*(6825), 144.

36. Vaux, D. (2012). Research methods: Know when your numbers are significant. *Nature*, *492*(7428), 180–181.

37. Godecharle, S., Nemery, B., & Dierickx, K. (2013). Integrity training: Conflicting practices. *Science*, *340*(6139), 1403.

38. McNutt, M. (2014). Reproducibility. *Science*, *343*(6168), 229.

39. Bradbury, A., & Plückthun, A. (2015). Standardize antibodies used in research. *Nature*, *518*(7537), 27–29.

40. McNutt, M. (2016). Implicit bias. *Science*, *352*(6289), 1035.

41. Reardon, S. (2016). A mouse’s house may ruin experiments. *Nature*, *530*(7590), 264.

42. Giles, J. (2007). Breeding cheats. *Nature*, *445*(7125), 242–243.

43. Sarewitz, D. (2012). Beware the creeping cracks of bias. *Nature*, *485*(7397), 149.

44. Alberts, B. (2015). Self-correction in science at work. *Science*, *348*(6242), 1421.

45. McNutt, M., Lehnert, K., Hanson, B., Nosek, B. A., Ellison, A. M., & King, J. L. (2016). Liberating field science samples and data. *Science*, *351*(6277), 1024–1026.

46. (2011). Copy and paste. *Nature*, *473*(7348), 419–420.

47. Marcus, A., & Oransky, I. (2011). The paper is not sacred. *Science*, *480*(2011), 449–450.

48. (2015). It’s good to talk. *Nature*, *523*(7561), 382.

49. McNutt, M., Lehnert, K., Hanson, B., Nosek, B. A., Ellison, A. M., & King, J. L. (2016). Liberating field science samples and data. *Science*, *351*(6277), 1024–1026.

50. (2015). Let’s think about cognitive bias. *Nature*, *526*(7572), 163.

51. DuBois, J., Chibnall, J. T., Tait, R., & Wal, J. V. (2016). Misconduct: Lessons from researcher rehab. *Nature*, *534*(7606), 173–175.
